# Breast cancer-associated SNP rs72755295 is a *cis*-regulatory variation for human *EXO1*

**DOI:** 10.1590/1678-4685-GMB-2021-0420

**Published:** 2022-10-10

**Authors:** Qiang Shi, Xing-Yuan Yao, Hong-Yan Wang, Ya-Jie Li, Xin-Xin Zhang, Chang Sun

**Affiliations:** 1 Shaanxi Normal University, College of Life Sciences, Xi’an, Shaanxi, P.R. China

**Keywords:** Breast cancer, EXO1, rs72755295, rs4149909, expression regulation

## Abstract

Breast cancer is the most common malignant tumor in women. A previous genome-wide association study reports that rs72755295, a SNP locating at intron of *EXO1* (exonuclease 1), is associated with breast cancer. Due to the complete linkage disequilibrium between rs72755295 and rs4149909, a nonsynonymous mutation for *EXO1*, rs4149909 is supposed to be the causal SNP. Since *EXO1* is overexpressed in breast carcinoma samples, we hypothesized that the genetic variations in this locus might confer breast cancer risk by regulating *EXO1* expression. To substantiate this, a functional genomics study was performed. The dual luciferase assay indicated that G of rs72755295 presents significantly higher relative enhancer activity than A, thus verifying that this SNP can influence gene expression in breast cell. Through chromosome conformation capture it was disclosed that the enhancer containing rs72755295 can interact with the *EXO1* promoter. RNA-seq analysis indicated that *EXO1* expression is dependent on the rs72755295 genotype. By chromatin immunoprecipitation, the transcription factor PAX6 (paired box 6) was recognized to bind the region spanning rs72755295. In electrophoretic mobility shift assay, G of rs72755295 displays obviously higher binding affinity with nuclear protein than A. Our results indicated that rs72755295 is a *cis*-regulatory variation for *EXO1* and might confer breast cancer risk besides rs4149909.

## Introduction

Breast cancer is the most common malignant tumor and one of the most important causes of cancer-related mortality among women worldwide (Sung et al., 2021). The predisposing factors of breast cancer can be divided into many environmental risk factors, including alcohol intake, obesity, endogenous hormone exposure and physical inactivity, and genetic susceptibility (Monninkhof et al., 2007, 2009; Lynch et al., 2011; Rojas and Stuckey, 2016; Huang et al., 2019). To disclose the potential genetic contribution for breast cancer, many genome-wide association studies (GWAS) have been carried out for this disease (see GWAS catalog at https://www.ebi.ac.uk/gwas/ for detail). In one GWAS, a genetic marker in *EXO1* (exonuclease 1) intron region, rs72755295, was identified to be associated with breast cancer in Caucasians (Michailidou et al., 2015). Due to the fact that this SNP is in strong linkage disequilibrium (LD) with a missense SNP in *EXO1*, rs4149909 (p.Asn279Ser), and that *EXO1* is suggested to be an oncogene (Liberti and Rasmussen, 2004; Keijzers et al., 2018), the GWAS signal in this locus is proposed to result from rs4149909 (Michailidou *et al.*, 2015). On the other hand, *EXO1* expression is observed to be significantly elevated in breast tumor tissue (Muthuswami et al., 2013; Qi et al., 2019; Liu and Zhang, 2021), hinting that the *cis*-regulatory variations for *EXO1* might also contribute to breast cancer risk. However, this issue has hardly been surveyed. 

In the current study, we hypothesized that the genetic variations in this locus, i.e., rs72755295, rs4149909 and/or other SNP(s) in LD with them, might have the ability to regulate *EXO1* expression. Functional genomics approach was used to investigate this possibility. 

## Material and Methods

### 1000 Genomes project data analysis

The genotype of +/-100 kb region surrounding rs72755295 was retrieved for all 26 populations from the 1000 Genomes project public dataset (http://www.internationalgenome.org/). The LD pattern was determined by ldSelect (Carlson et al., 2004) or Genome Variation Server (http://gvs.gs.washington.edu/GVS150/) with *r* ^*2*^ threshold as 0.8.

### Plasmid construct and mutagenesis

PCR primers were designed by primerselect 7.0 (DNASTAR Inc, Madison, WI) and synthesized (Sangon Biotech, Shanghai, China). rs72755295 and rs4149909 surrounding regions (**~**1.5 kb) were amplified with Q5 High-Fidelity DNA Polymerase (NEB, Ipswich, MA) and primers shown in Table S1. Thermocycling conditions for routine PCR was as follows: 98 ℃ for 30 s; 35 cycles of 98 ℃ for 10 s, 68 ℃ for 30 s, 72 ℃ for 45 s, and finally 72 ℃ for 2 min. The PCR product and pGL3-promoter vector (Promega, Madison, WI) were digested by *Mlu*I and *Xho*I (NEB), purified by GeneJET Gel Extraction Kit (Thermo Fisher Scientific) and ligated by T4 DNA ligase (NEB) according to the manufacturer’s manual. The recombinant plasmids were transformed into *E.coli* DH5α competent cells (Takara, Dalian, China), cultured, and then extracted by TIANpure Midi Plasmid Kit (Tiangen Biotech, Beijing, China). After sequencing, the plasmids with corresponding alleles were generated by Q5 Site-Directed Mutagenesis Kit (NEB) and primers in Table S1. Before transfection, all plasmids were sequenced to rule out artificial mutations and verify the haplotype orientation.

## Cell culture, transient transfection and Dual Luciferase Reporter Gene Assay

Human breast cancer cell line MCF-7 was cultured in DMEM (High Glucose, Biological Industries, Cromwell, CT) with 10% fetal bovine serum (Biological Industries) and 1% penicillin-streptomycin solution (Solarbio, Beijing, China) and incubated in 5% CO_2_ at 37 ℃. MCF-7 cells were seeded into 24-well plate at density 1.0 × 10^4^ cells/well and transfected after 24 hours. The recombinant plasmid DNA (475 ng) was transfected into MCF-7 cells using Lipofectamine 2000 (Thermo Fisher Scientific) according to the manufacturer’s recommendation. Cells were harvested and lysed by passive lysis buffer (Promega) after 36 hours culture. Co-transfection of *Renilla* luciferase reporter (pRL-TK, 25 ng, Promega) plasmid was performed as an internal control along with the recombinant plasmid. Luciferase activity was read by the Dual-Luciferase Reporter Assay System (Promega) using GloMax Navigator (Promega) with a costar® 96-well white polystyrene plate according to the manufacturer’s protocol. The relative enhancer activity was expressed as the ratio between firefly and *Renilla* luciferase and the empty pGL3-promoter vector was utilized as the negative control. Six independent replicates were carried out for each experiment.

### Chromosome conformation capture (3C)

3C technology was utilized to detect the long-distance interaction between enhancer and promoter of nearby genes and the ligation frequency was quantified by real-time PCR. Generally, ~10^8^ MCF-7 cells in the logarithmic growth phase were detached with 0.25% Trypsin-0.02% EDTA Solution (Solarbio) and harvested into a 50 mL Conical Sterile Polypropylene Centrifuge Tube (Corning life sciences, Tewksbury, MA). MCF-7 cells were resuspended in 10 ml DMEM medium and cross-linked with 278 μl 37% formaldehyde (1% final concentration) for shaking 10 min at room temperature. The cross-linking reaction was terminated by adding 500 μl 2.5 M glycine (0.125 M final concentration) and incubated for 15 min on ice. After centrifugation, cells were lysed with lysis buffer containing protease inhibitor cocktail (Sigma, St. Louis, MO). The chromatin was digested by *Bgl*II (1000 units, NEB) at 37 ℃ for 12 hours with 900 rpm shaking and the digestion products were assessed by 0.8% agrose gel electrophoresis. After ligation by high concentration T4 DNA ligase (10000 units; NEB), the products were treated overnight with 15 μl proteinase K (20 mg/ml; Sigma) at 65 ℃ with 300 rpm shaking. After 30 μl RNase A (10 mg/ml; Takara) treatment at 37 ℃ for 45 min, DNA was isolated by the phenol-chloroform method.

The BAC (bacterial artificial chromosome) RP11-610O24 containing partial 1q43 region was obtained from BACPAC Resources Center (http://bacpac.chori.org/), cultured in LB medium supplemented with chloramphenicol, extracted by the Large-Construct Kit (Qiagen, Valencia, CA), digested by *Bgl*II (NEB) as abovementioned, ligated as control and also recovered by the phenol-chloroform method.

The relative amount of 3C product was measured by real-time PCR in a CFX96TM Real-time Detection System (Bio-Rad, Hercules, CA) with unidirectional primers listed in Table S2. The enrichment for MCF-7 cells relative to BAC was calculated using the comparative Ct method. All 3C PCR products were verified by resequencing.

### RNA-seq analysis

The RNA-seq data (sra format) for lymphoblastoid cell lines (LCL; Montgomery et al., 2010; Jadhav et al., 2019) was obtained from the SRA database (https://www.ncbi.nlm.nih.gov/sra/) and converted into fastq format by SRA toolkit (https://github.com/ncbi/sra-tools). After alignment with the *EXO1* mRNA sequence by bowtie2 (Langmead and Salzberg, 2012), the expression was calculated by RSEM (Li and Dewey, 2011) with default parameter and reported as FPKM (Fragments Per Kilobase of transcript per Million fragments mapped). The genotypes for LCLs were obtained from the 1000 Genomes or HapMap project.

### Chromatin immunoprecipitation (ChIP)

TRANSFAC database (http://www.gene-regulation.com/) was used to predict potential transcription factors (TF). ChIP was performed in MCF-7 cell lines by EZ ChIP Kit (Millipore, Burlington, MA) according to the manufacturer’s instruction. In brief, formaldehyde (1% final concentration) was used to cross-link the proteins to the DNA for 10 min at 25 ℃ in ~10^7^ cells. Glycine (0.125 M final concentration) was added to quench the formaldehyde and terminate the cross-linking reaction. Cells were rinsed twice with 10 mL cold PBS, scraped thoroughly with a cell scraper, transferred into 50 mL tube and centrifuged at 1000 g for 5 min at 4 ℃. Cell pellets were resuspend in ChIP lysis buffer and incubated for 10 min on ice. Cells lysates were sonicated to shear DNA into an average fragment size of 200-1000 bp by Ultrasonic Homogenizer (Scientz Biotechnology, Ningbo, China) and the fragment sizes were analyzed on a 1.5% agarose gel. Chromatin samples were diluted with 10-fold dilution buffer, and precleared with protein A beads for 1 h at 4 ℃. For immunoprecipitation, the sheared chromatin was incubated with related mouse antibodies or normal mouse IgG (Santa Cruz Biotechnology, Santa Cruz, CA) at 4 ℃ overnight, respectively, and precipitated with 5000 g for 1 min at 4 ℃ by protein A beads. The immunoprecipitated protein/chromatin complex was washed as follows: once by low salt, high salt, LiCl wash buffer and twice by TE buffer. After washing, the protein/chromatin complex was resuspended in elution buffer. Cross-linking was reversed and protein was digested by proteinase K (Sigma). DNA was purified using GeneJET Gel Extraction Kit (Thermo Fisher Scientific) and quantified by real-time PCR to assess the enrichment by iQ SYBR green (Bio-Rad) and primer pair ACAGTTGCCAGTAGTAGTCTTTTA and TCTCATATCATCCTAGCCAACAAT.

### Electrophoretic mobility shift assay (EMSA)

Nuclear proteins were isolated from human MCF-7 cells using Nuclear and Cytoplasmic Protein Extraction Kit (Beyotime, Shanghai, China) and protein concentration was measured in an Epoch Microplate Spectrophotometer (BioTek, Winooski, VT) with Enhanced BCA Protein Assay Kit (Beyotime). The probes for both alleles of rs72755295 listed in Table S3 were labeled with 3′-Biotin by EMSA probe biotin labeling Kit (Beyotime). In brief, 10 fmols of the biotin-labeled probes were incubated with 5 μg of nuclear proteins for 20 min at room temperature. The DNA-protein complexes were run on a 4.9% native polyacrylamide gel, transferred to a positively charged nylon membrane (Beyotime) and cross-linked by UV-light. For each electrophoresis, the biotin-labeled probes without nuclear protein and probe-protein complex incubating with competitor oligonucleotides (non-labeled probes) were also included as controls. After blocking and incubating with streptavidin-HRP (horseradish peroxidase) conjugate, the membrane was visualized by chemiluminescent EMSA kit (Beyotime) according to the manufacturer’s protocol. The 8-bit images of EMSA signals were captured by a Luminescent Imaging Workstation (Tanon, Shanghai, China).

### Statistics

Student’s *t*-test was performed by SPSS 20.0 (IBM, Armonk, NY) to evaluate the luciferase expression difference among plasmid constructs, the enrichment for 3C and ChIP and *EXO1* expression between different genotype groups. The null hypothesis was rejected when *P*< 0.05.

## Result

### Genetic variations nearby rs72755295

Within the 200 kb region surrounding rs72755295, there are ~1200 SNPs in each population and two distinct LD patterns could be observed. In all populations from Europe, South Asia and America, only one variation, rs4149909 (~10.4 kb away from rs72755295), shows complete LD (*r* ^*2*^ =1) with rs72755295 and the minor allele (G for both SNPs) frequency varies from 1% to 5% (see Table S4), which is consistent with previous observation in Caucasians (Michailidou et al., 2015). All other SNPs present a relatively low LD with rs72755295 (all *r* ^*2*^ <0.11; result not shown). In contrast, in all populations from East Asia and Africa, these two SNPs are not in polymorphism (see Table S4).

### Function of rs72755295 and rs4149909 in regulating gene expression

**Figure 1 -:**
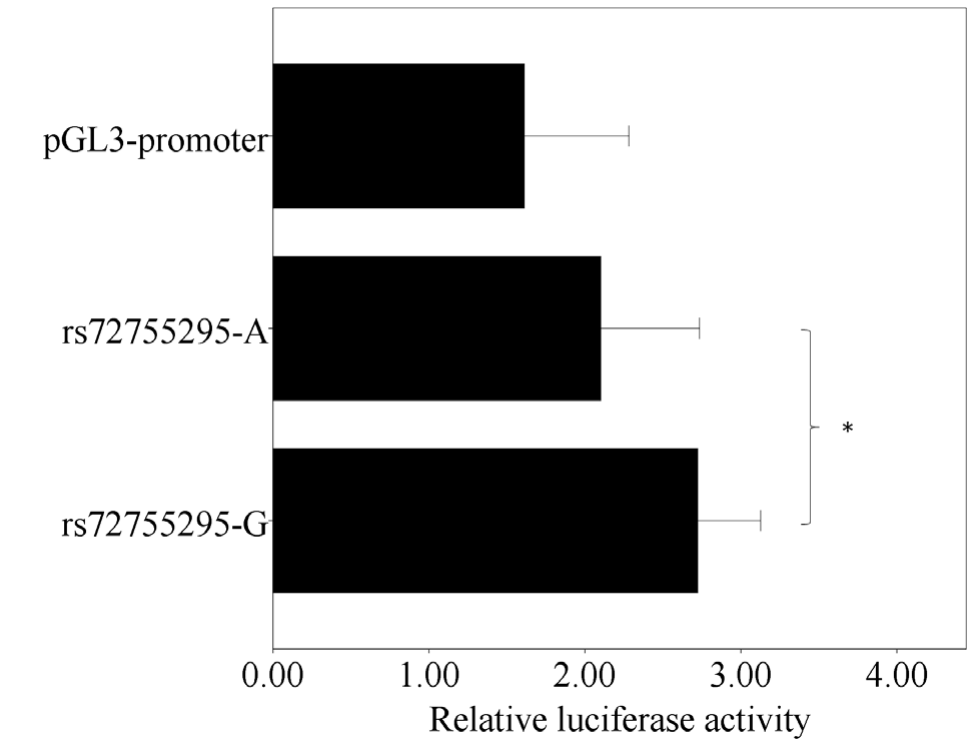
Relative luciferase activity for different rs72755295 alleles in MCF-7 cell. The *x* axis represents relative enhancer activity. All data are expressed as mean ± standard deviation (SD). * indicates *P*<0.01.

To investigate the role of the two SNPs on gene expression regulation, we constructed a luciferase plasmid containing the surrounding region of these two SNPs and generated the plasmid with another allele by mutagenesis. For rs4149909, no significant difference was observed in luciferase activity between the A and G allele (*P*=0.22; Figure S1), indicating that rs4149909 does not have the function to alter gene expression. In contrast, the G allele of rs72755295 shows ~29.6% higher relative luciferase activity than A (*P*=0.0022; Figure 1), which indicates that rs72755295 is a functional site and can regulate gene expression in breast cells. rs72755295 is located in an intron region of *EXO1* and not within the promoter of any known gene. Moreover, there are multiple H3K27Ac and H3K4me1 peaks nearby rs72755295 in human mammary epithelial cell (Figure S2), two frequent histone modifications in active enhancer (Calo and Wysocka, 2013). Therefore, it is reasonable to hypothesize that rs72755295 is within an enhancer region and can alter enhancer activity.

### Interaction between EXO1 promoter and the enhancer containing rs72755295

**Figure 2-:**
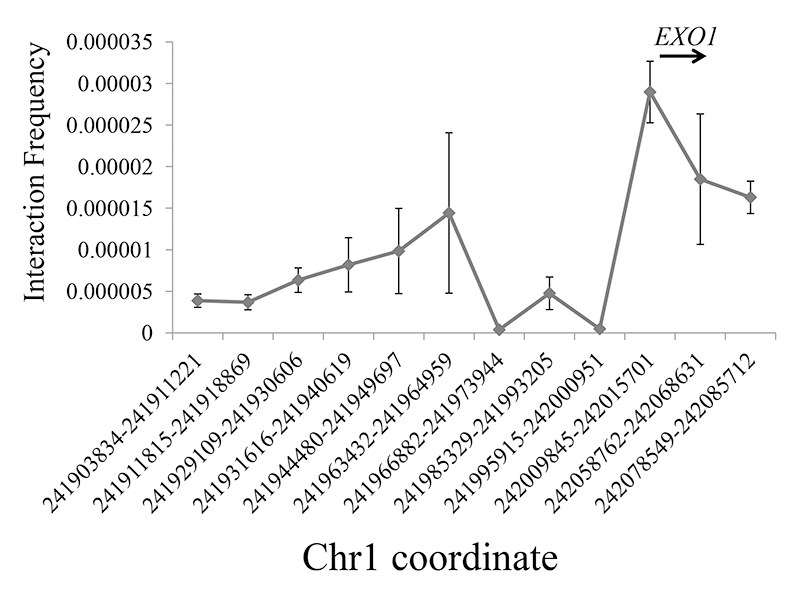
Interaction efficiency between the enhancer containing rs72755295 and surrounding genome regions in 1q43. The *x* axis indicates the location of restriction fragments in chr1 (relative to human genome build 37) while the *y* axis shows the relative interaction efficiency. The above arrow shows the schematic *EXO1* position and transcript direction. All data is shown as mean±SD.

Given that rs72755295 is within an enhancer region, remains unclear whether its target gene is *EXO1*. 3C was utilized to examine whether the enhancer region could physically interact with the *EXO1* promoter. In our assay, the constant primer was set in the enhancer containing rs72755295 while the anchoring primers were set in the *EXO1* promoter and eleven random regions (Table S2). As shown in Figure 2, a strong ligation frequency was detected in the *EXO1* promoter region (corresponding to 10^th^ point in *x*-axis, **~**24.7 kb away from the enhancer). A one-sample *t*-test utilized to compare the ligation frequency between *EXO1* promoter and other regions in our assay, revealed a significant deviation (*P*<10^-6^), thus suggesting that *EXO1* should be the regulation target of this enhancer in breast cells.

### Association between rs72755295 genotype and EXO1 expression

**Figure 3-:**
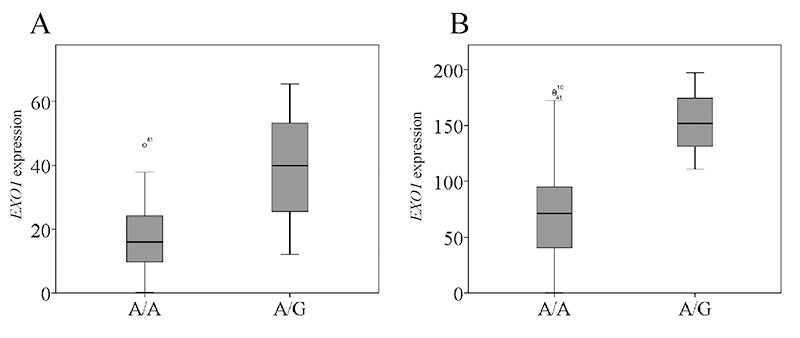
Relationship between rs72755295 genotype and *EXO1* expression in LCL at CEU population from literature Jadhav *et al.* (2019) (A) and Montgomery *et al*. (2010) (B). The expression is displayed as FPKM.

If rs72755295 can indeed influence *EXO1* expression, this SNP should be an expression quantitative trait locus (eQTL) for this gene. To verify this issue, RNA-seq data from LCL, a well-established model for eQTL analysis, were obtained from the literature (Montgomery et al., 2010; Jadhav et al., 2019) and *EXO1* expression was calculated. Since A is fixed for rs72755295 in CHS (Southern Han Chinese) and YRI (Yoruba in Ibadan, Nigeria; see Table S4) populations, only their CEU (Utah Residents with Northern and Western European Ancestry) data were included for analysis (Jadhav *et al.*, 2019). In both CEU datasets, no individuals are homozygous for the G allele at rs72755295 due to the low frequency. Therefore, an independent *t*-test was utilized to compare the *EXO1* expression between the A/A and A/G group. As shown in Figure 3A, the average *EXO1* expression is ~136.9% higher in the A/G group than in A/A (*P*=0.001) for the dataset from literature (Jadhav *et al.*, 2019). A similar result was obtained for the data from another literature (*P*=0.009; Figure 3B; Montgomery *et al.*, 2010), which is consistent with our luciferase result and confirms that rs72755295 is an eQTL for *EXO1*.

### Related TF binding rs72755295

**Figure 4 -:**
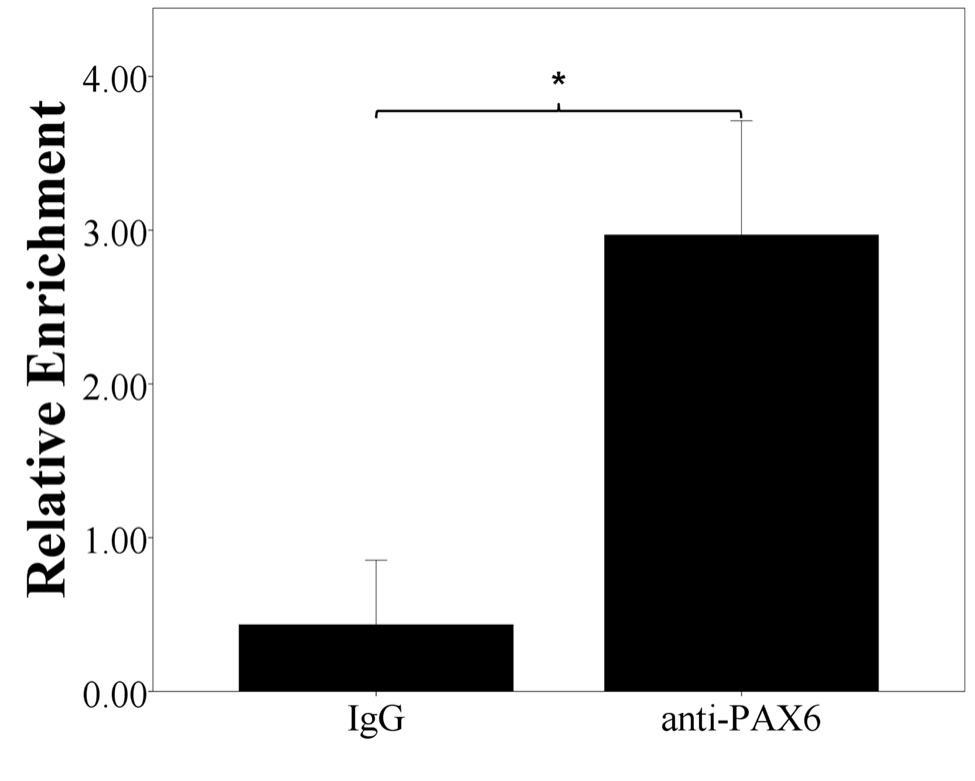
Enrichment of the chromatin spanning rs72755295 in MCF-7 cell line. The *y* axis represents relative enrichment. The result is normalized by input and the data is expressed as mean±SD. * indicates *P*<0.001.

Based on the fact that rs72755295 is located in an enhancer, it seems that it might interact with TF and might influence TF binding affinity. The prediction by Match at TRANSFAC indicated that rs72755295 might be within a binding site of PAX6 (paired box 6) and could alter the binding affinity of this transcription factor. To verify this prediction, ChIP was carried out with related antibody in MCF-7 cell line and real-time PCR was used to evaluate the relative chromatin enrichment. As shown in Figure 4, compared with IgG, the region containing rs72755925 is immunoprecipated by the PAX6 antibody (*P*=0.00050), thus confirming that PAX6 can bind the rs72755925 surrounding region in MCF-7.

### TF binding affinity difference between rs72755295 alleles

**Figure 5 -:**
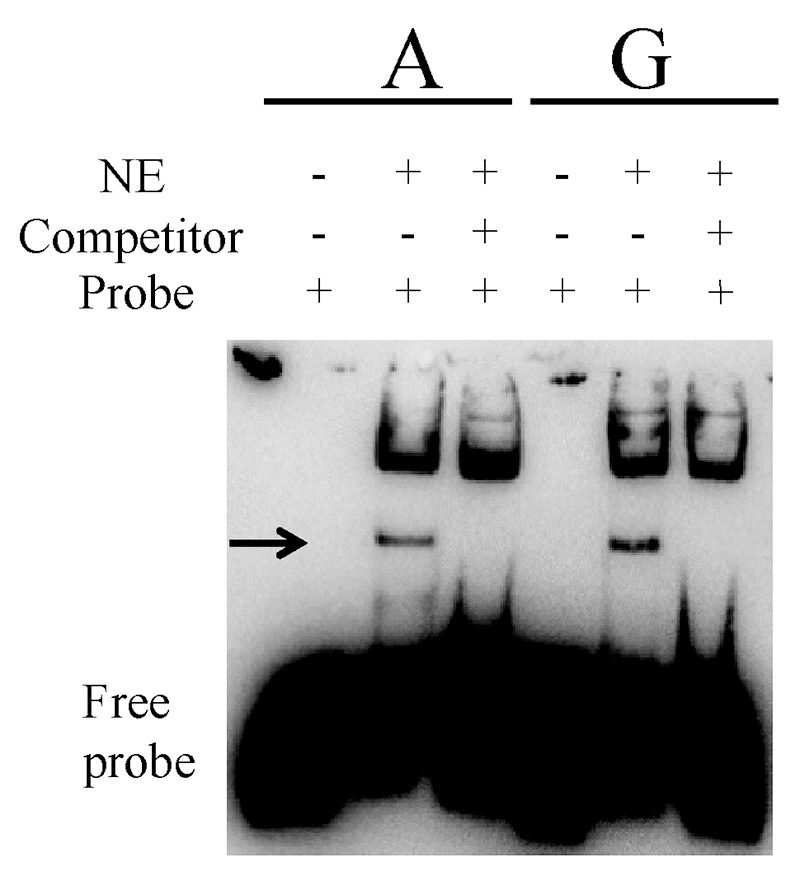
Difference in the binding affinity between MCF-7 nuclear proteins and rs72755295 alleles in EMSA. The top line indicates different alleles. NE denotes nuclear protein, and the arrow points out the position of protein-probe complex.

To verify the binding capacity difference between rs72755295 alleles, EMSA was performed with nuclear extract prepared from MCF-7 cells. It can be observed that there is a specific protein-DNA complex band composed of the core sequence containing rs72755295 and nuclear proteins (Figure 5). Moreover, the G allele of rs72755295 shows an apparently higher binding affinity with nuclear protein than the A allele (see Figure 5), which is consistent with our luciferase result. 

## Discussion

In the present research, population genetics and functional genomics approaches were utilized to explore the potential *cis*-regulatory variations for *EXO1*, which might further contribute to breast cancer predisposition. To achieve this goal, the 1000 Genomes project data were recruited and the LD pattern was surveyed in this locus. As a result, only rs4149909 was identified to be in complete LD with rs72755295 in multiple populations. Further luciferase and ChIP assays verified that only rs72755295 can regulate gene expression in breast tissue by altering the binding affinity of the transcript factor PAX6. By 3C, the target gene, *EXO1*, was disclosed for this enhancer. Our effort provides more insight into the expression regulation of *EXO1*. 

*EXO1*, locating at chromosome 1q42-43, has 14 exons spanning over ~41.7 kb and yields a **~**3 kb mRNA transcript (Tishkoff et al., 1998). EXO1 is a 5’ to 3’ exonuclease protein (Schmutte et al., 1998; Lee and Wilson, 1999) and also with ability of 3’-5’ exonucleolytic degradation of DNA (Genschel et al., 2002), thus playing an essential role in DNA repair, replication and recombination (Qiu et al., 1999; Tran et al., 2004; Mimitou and Symington, 2008; Nimonkar et al., 2011). The link between *EXO1* and cancer is intriguing and usually interpreted by two distinct models (Liberti and Rasmussen, 2004). The deficiency of EXO1 activity induced by germline mutation can lead to the inactivation of DNA mismatch repair pathway, hypermutation in genome and further predispose the carriers to develop cancer (Liberti and Rasmussen, 2004; Keijzers et al., 2016). This model has been verified by the observation in human hereditary nonpolyposis colorectal cancer (Wu et al., 2001) and mouse model with *EXO1* knockout (Wei et al., 2003; Bardwell et al., 2004; Schaetzlein et al., 2013). Alternatively, a higher EXO1 expression or activity will induce the increase of recombination rate, impaired repair of DNA double-strand breaks, telomere resection and activation of Ras/PI3K signaling pathway, which may also further increase the cancer susceptibility (Liberti and Rasmussen, 2004; Muthuswami et al., 2013). In the case of breast, our results indicate that the risk allele, G of rs72755295 (Michailidou et al., 2015), can cause a higher *EXO1* expression. Moreover, an increased expression of *EXO1* has been frequently observed in tumor tissues compared with normal breast ones (Kretschmer et al., 2011; Muthuswami *et al.*, 2013; Qi *et al.*, 2019; Saha et al., 2019; Liu and Zhang, 2021). All these results hint that the latter model may play a more important role in the association between rs72755295 and breast cancer. An *EXO1* overexpression assay or genome editing on this locus and followed by cell function investigation will shed more light on the effect of rs72755295 in tumorigenesis.

Our eQTL analysis indicated that *EXO1* expression is dependent on the genotype of rs72755295. To further validate this issue, we searched the GTEx Portal database (https://gtexportal.org/; GTEx Consortium, 2017) but no association was observed (result not shown). This might be due to the relatively low frequency of rs72755295 G allele, which could decrease the power of statistical testing. In addition, the potential correlation might be influenced by some environmental or physiological effects as suggested (Gagneur et al., 2013).

Our result suggests that the *cis*-regulation of rs72755295 on *EXO1* expression is dependent on PAX6 in breast cells. To further validate this issue, we downloaded RNA-seq data for breast tissues (Wenric et al., 2017), calculated *EXO1* and *PAX6* expression as described above and performed a correlation analysis. As shown in Figure S3, there is a significant correlation between *EXO1* and *PAX6* expression (*r*=0.528, *P*=0.0046), which is consistent with our conclusion. It is also useful to compare the correlation between A/A and A/G group. However, due to the small sample size of the A/G group, the comparison might not be with enough power to display the binding affinity difference. Moreover, it is interesting to observe that the knockdown of *PAX6* can remarkably inhibit cell viability, DNA synthesis and colony formation in breast cancer cell line and tumorigenesis in xenograft nude mice (Zong et al., 2011). Considering the role of EXO1, it might be proposed that PAX6 plays this role, at least partially, through *trans*-regulation of *EXO1.*

Besides breast cancer, this locus is also suggested to be associated with pancreas (Dong et al., 2011), colon (Madi et al., 2018) and keratinocyte (Liyanage et al., 2019) cancer. Interestingly, *EXO1* overexpression in tumor cells compared with corresponding normal ones is also observed in multiple human tissues, including liver (Dai et al., 2018; Yang et al., 2020), lung (Zhou et al., 2021), pancreas and colon (Rasmussen et al., 2000). Moreover, a database search through UALCAN (http://ualcan.path.uab.edu/index.html; Chandrashekar et al., 2017) confirms that significant *EXO1* overexpression in cancer cell is appearing in almost all tissue types from TCGA (The Cancer Genome Atlas) project (results not shown). Considering this and the ubiquitous spread of EXO1 and PAX6 (see http://biogps.org) in human tissues, it might be proposed that the putative enhancer and rs72755295 might be also involved in the carcinogenesis in abovementioned tumor types, which deserves further investigation.

Platinum salts have been widely utilized in chemotherapy of multiple human cancer types and act through crosslinking with DNA, causing DNA damage and further inducing cancer cell apoptosis (Dasari and Tchounwou, 2014). EXO1 can excise the adducted nucleotide and mediate DNA repair, which might lead to resistance in platinum salts treatment. Therefore, a lower *EXO1* expression will be beneficial to cancer patients in platinum salts treatment, which has been validated in ovarian cells (Zhou et al., 2014; He et al., 2020). To validate this issue, we also searched TCGA data through EviCor database (https://www.evicor.org/; Petrov and Alexeyenko, 2022). For breast invasive carcinoma (BRCA) patients with lower *EXO1* expression, carboplatin treatment can promote patients' survival (see Figure S4). In contrast, for BRCA patients with higher *EXO1* expression, the same treatment presents a higher risk of death (*P*=0.0304; see Figure S4), which is consistent with a previous proposal (Zhou *et al.*, 2014; He *et al.*, 2020). Considering the role of rs72755295 on *EXO1* expression regulation, this SNP might contribute to the difference in platinum salts response among cancer patients, which has been preliminarily verified by a recent pharmocogenetics study in advanced colorectal cancer (Madi et al., 2018) and deserves further research. 

## Supplementary Material

The following online material is available for this article:

Table S1 - Primers used in plasmid construction and mutagenesis.

Table S2 - Primers used in 3C-qPCR.

Table S3 - Probes for rs72755295 in EMSA.

Table S4 -
*r* ^*2*^ between rs72755295 and rs44149909 and minor allele frequency in 1000 Genomes project populations.

Figure S1- Relative enhancer activity for different alleles of rs4149909.

Figure S2 - Histone modification for the region surrouding rs72755295 in breast cell.

Figure S3 - The correlation between *PAX6* and *EXO1* expression in breast tissues.

Figure S4 - Kaplan-Meier survival curves for BRCA patients grouped by *EXO1* expression and treatment.
