# Supplementary material for: Breast cancer-associated SNP rs72755295 is a cis-regulatory variation for human EXO1
Source: Genet Mol Biol. 2022 Oct 10;45(4):e20210420. doi: 10.1590/1678-4685-GMB-2021-0420 (PMC9631386; doi:10.1590/1678-4685-GMB-2021-0420)
Supplement: Table S1 - [file 1415-4757-GMB-45-4-e20210420-s1.pdf]

Genetics and Molecular Biology

https://doi.org/10.1590/1678-4685-GMB-2021-0420

Supplementary Material to "Breast cancer-associated SNP rs72755295 is a *cis*-regulatory variation for human *EXO1*"

Table S1 - Primers used in plasmid construction and mutagenesis.

	Primer for PCR	Annealing temp (°C)	Primer for mutagenesis[a]	Annealing temp (°C)
rs4149909	CAGTC-ACGCGT-AGGATCCGTTTGGTTCTCTGG	68	ATTCGGGCCAgCAATACCTTC	61
	CAGTC-CTCGAG-GTGTTTTCATAGGGTGGTCATCA		AAACCCGTTGATGTAATCC	
rs72755295	CAGTC-CTCGAG-CCYGGCCTGTACATGTTCTTTCA	68	CCATATCTACcGTATATTAGTTATGG	56
	CAGTC-ACGCGT-ATGGTTTATGTGTTCTGGGTTTTGA		CTTTGCATTGTTGGCTAG	

[a]The taget site in lower case.
