# Supplementary material for: Breast cancer-associated SNP rs72755295 is a cis-regulatory variation for human EXO1
Source: Genet Mol Biol. 2022 Oct 10;45(4):e20210420. doi: 10.1590/1678-4685-GMB-2021-0420 (PMC9631386; doi:10.1590/1678-4685-GMB-2021-0420)
Supplement: Table S2 - [file 1415-4757-GMB-45-4-e20210420-s2.pdf]

Genetics and Molecular Biology

https://doi.org/10.1590/1678-4685-GMB-2021-0420

Supplementary Material to "Breast cancer-associated SNP rs72755295 is a *cis*-regulatory variation for human *EXO1*"

Table S2 - Primers used in in 3C-qPCR.

Primer name	Primer sequence	Location in genome[a]	Restriction fragment location	Target element
Target primer 1	AGACACAGGAAGGAAAGACTTCCTGG	Chr1: 241911071-241911096	241903834-241911221	
Target primer 2	ATTCTGCATATTACCCCTTTGTCAGATAG	Chr1: 241918753-241918781	241911815-241918869	
Target primer 3	TCCTTCTCATTCACCACCTCAACAC	Chr1: 241930487-241930511	241929109-241930606	
Target primer 4	TCACTGTAATCTCCAACTCCTGCAAGT	Chr1: 241940469-241940495	241931616-241940619	
Target primer 5	CTTGCTTGCCCTCCTCCTTTTAG	Chr1: 241949599-241949621	241944480-241949697	
Target primer 6	GTCAGAACATGTGCAGTAAAAGGCAC	Chr1: 241964873-241964898	241963432-241964959	
Target primer 7	AATGTAAGCCAGGTATCAGAAAAGACAAG	Chr1: 241973842-241973870	241966882-241973944	
Target primer 8	GTAAACTGGCTAGGTAAAAAGTTAGTGTAAAATAAA	Chr1: 241993055-241993090	241985329-241993205	
Target primer 9	ACAAAGTAATTCCTAGCATCAGTAGGAGCC	Chr1: 242000842-242000871	241995915-242000951	
Target primer 10	CTCATGGGATCAAGCCTATTCTCGT	Chr1: 242015635-242015659	242009845-242015701	*EXO1* promoter
Target primer 11	TTGAGCAAGAAATGGTTAGTTAACCTGTG	Chr1: 242068577-242068605	242058762-242068631	
Target primer 12	TAGATAAGCCATGAAACAGTAATTTTTGCA	Chr1: 242085612-242085641	242078549-242085712	
Constant primer	TGCCTGCATTTTGGAAGCCTG	Chr1: 242040408-242040428	242032104-242040496	enhancer region

[a]Relative to human genome build37.
