# Supplementary material for: Breast cancer-associated SNP rs72755295 is a cis-regulatory variation for human EXO1
Source: Genet Mol Biol. 2022 Oct 10;45(4):e20210420. doi: 10.1590/1678-4685-GMB-2021-0420 (PMC9631386; doi:10.1590/1678-4685-GMB-2021-0420)
Supplement: Table S3 - [file 1415-4757-GMB-45-4-e20210420-s3.pdf]

Genetics and Molecular Biology

https://doi.org/10.1590/1678-4685-GMB-2021-0420

Supplementary Material to "Breast cancer-associated SNP rs72755295 is a *cis*-regulatory variation for human *EXO1*"

Table S3 - Probes for rs72755295 in EMSA.

SNP	Allele	Primer sequence[a]
rs72755295	A allele	ATAACTAATATACAGTAGATATGGCTT
		AAGCCATATCTACTGTATATTAGTTAT
	G allele	ATAACTAATATACGGTAGATATGGCTT
		AAGCCATATCTACCGTATATTAGTTAT

[a]The target sites underlined.
