# Supplementary material for: Breast cancer-associated SNP rs72755295 is a cis-regulatory variation for human EXO1
Source: Genet Mol Biol. 2022 Oct 10;45(4):e20210420. doi: 10.1590/1678-4685-GMB-2021-0420 (PMC9631386; doi:10.1590/1678-4685-GMB-2021-0420)
Supplement: Table S4 - [file 1415-4757-GMB-45-4-e20210420-s4.pdf]

*Genetics and Molecular Biology*

https://doi.org/10.1590/1678-4685-GMB-2021-0420

# Supplementary Material to "Breast cancer-associated SNP rs72755295 is a *cis*-regulatory variation for human *EXO1*"

**Table S4** - $r^2$ between rs72755295 and rs44149909 and minor allele frequency in 1000 Genomes project populations.

| Code | Population | $r^2$ | Minor allele frequency (%) |
|---|---|---|---|
| CHB | Han Chinese in Beijing, China | -[a] | - |
| JPT | Japanese in Tokyo, Japan | - | - |
| CHS | Southern Han Chinese | - | - |
| CDX | Chinese Dai in Xishuangbanna, China | - | - |
| KHV | Kinh in Ho Chi Minh City, Vietnam | - | - |
| CEU | Utah Residents (CEPH) with Northern and Western European Ancestry | 1 | 5 |
| TSI | Toscani in Italia | 1 | 3 |
| FIN | Finnish in Finland | 1 | 4 |
| GBR | British in England and Scotland | 1 | 5 |
| IBS | Iberian Population in Spain | 1 | 3 |
| YRI | Yoruba in Ibadan, Nigeria | - | - |
| LWK | Luhya in Webuye, Kenya | - | - |
| GWD | Gambian in Western Divisions in the Gambia | - | - |
| MSL | Mende in Sierra Leone | - | - |
| ESN | Esan in Nigeria | - | - |
| ASW | Americans of African Ancestry in SW USA | - | - |
| ACB | African Caribbeans in Barbados | - | - |
| MXL | Mexican Ancestry from Los Angeles USA | 1 | 2 |
| PUR | Puerto Ricans from Puerto Rico | 1 | 4 |
| CLM | Colombians from Medellin, Colombia | 1 | 2 |
| PEL | Peruvians from Lima, Peru | - | - |
| GIH | Gujarati Indian from Houston, Texas | 1 | 1 |
| PJL | Punjabi from Lahore, Pakistan | 1 | 1 |
| BEB | Bengali from Bangladesh | 1 | 1 |
| STU | Sri Lankan Tamil from the UK | 1 | 2 |
| ITU | Indian Telugu from the UK | 1 | 1 |

[a]Not in polymorphism.
