# Supplementary material for: Breast cancer-associated SNP rs72755295 is a cis-regulatory variation for human EXO1
Source: Genet Mol Biol. 2022 Oct 10;45(4):e20210420. doi: 10.1590/1678-4685-GMB-2021-0420 (PMC9631386; doi:10.1590/1678-4685-GMB-2021-0420)
Supplement: Figure S1- [file 1415-4757-GMB-45-4-e20210420-s5.pdf]

https://doi.org/10.1590/1678-4685-GMB-2021-0420

Supplementary Material to "Breast cancer-associated SNP rs72755295 is a *cis*-regulatory variation for human *EXO1*"

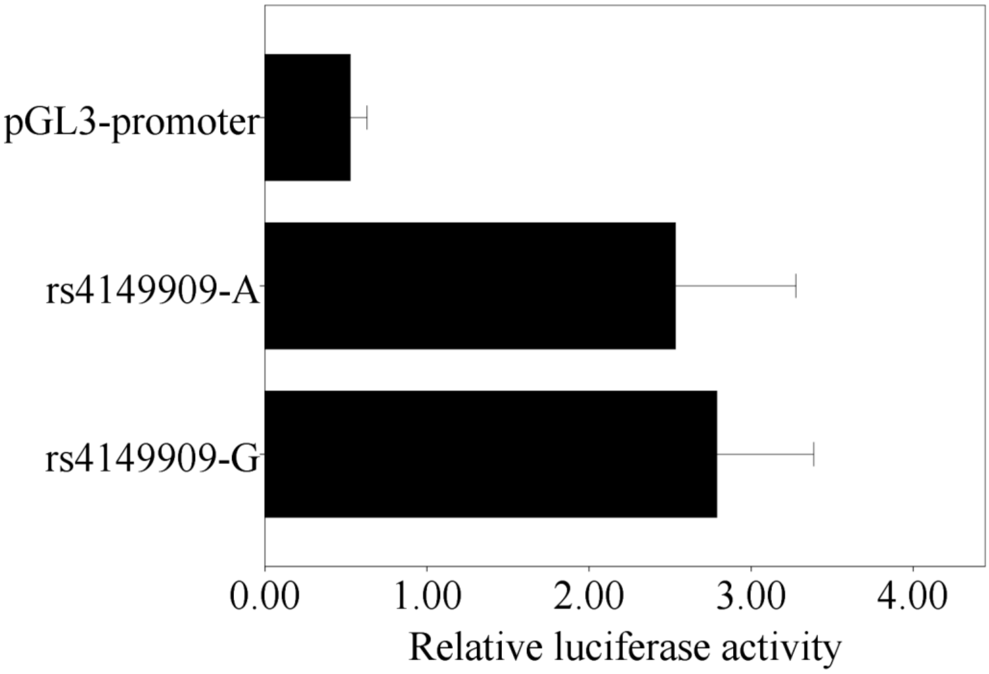

Figure S1 - Relative enhancer activity for different alleles of rs4149909. The *x* axis indicates relative luciferase activity. All data are expressed as mean ± standard deviation (SD).
