# Supplementary material for: Breast cancer-associated SNP rs72755295 is a cis-regulatory variation for human EXO1
Source: Genet Mol Biol. 2022 Oct 10;45(4):e20210420. doi: 10.1590/1678-4685-GMB-2021-0420 (PMC9631386; doi:10.1590/1678-4685-GMB-2021-0420)
Supplement: Figure S2 - [file 1415-4757-GMB-45-4-e20210420-s6.pdf]

Genetics and Molecular Biology

https://doi.org/10.1590/1678-4685-GMB-2021-0420

Supplementary Material to "Breast cancer-associated SNP rs72755295 is a *cis*-regulatory variation for human *EXO1*"

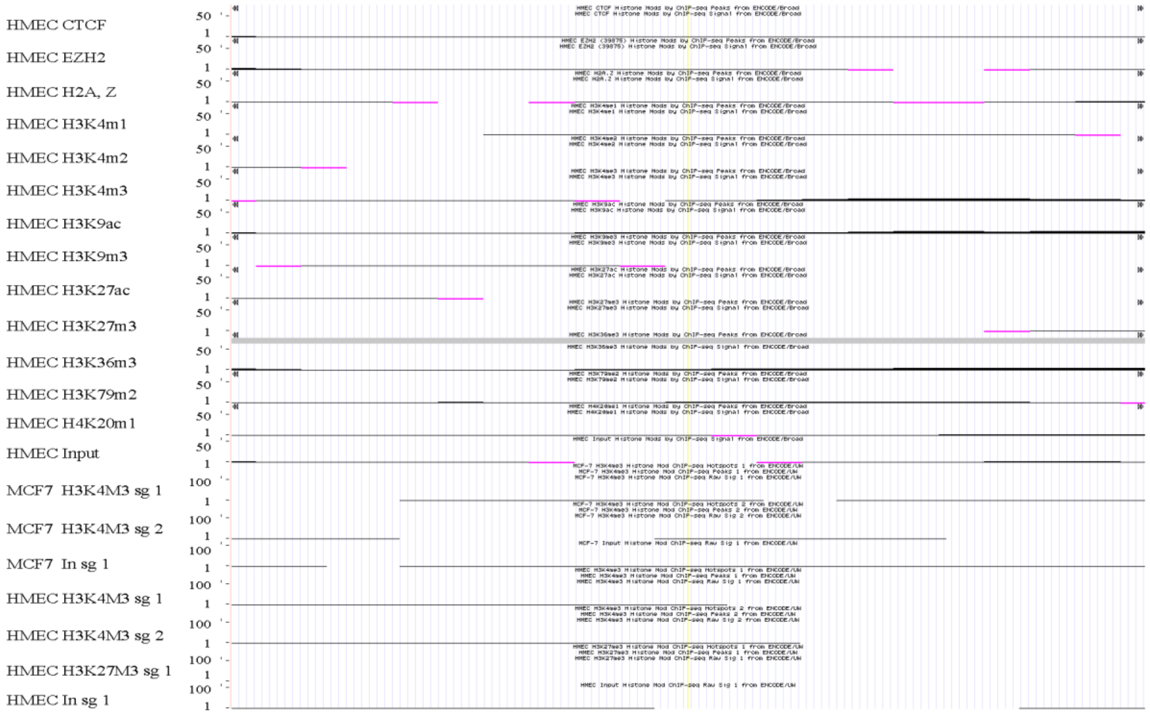

Figure S2 - Histone modification for the region surrouding rs72755295 in breast cell. The yellow line in middle indicates the position of rs72755295. Each part represents one kind of histone modification. The *x* axis indicates genome coordinate in chromosome 1. The *y* axis denotes the signal magnitude of ChIP-seq.
